# Main clinical features in patients at their first psychiatric admission to Italian acute hospital psychiatric wards. The PERSEO study

**DOI:** 10.1186/1471-244X-7-3

**Published:** 2007-01-19

**Authors:** Andrea Ballerini, Roberto M Boccalon, Giancarlo Boncompagni, Massimo Casacchia, Francesco Margari, Lina Minervini, Roberto Righi, Federico Russo, Andrea Salteri, Sonia Frediani, Andrea Rossi, Marco Scatigna, the PERSEO study group

**Affiliations:** 1 Servizio Psichiatrico Diagnosi e Cura, Santa Maria Nuova Hospital, Firenze, Italy; 2 Servizio Psichiatrico Diagnosi e Cura, Sant' Anna Hospital, Ferrara, Italy; 3 Servizio Psichiatrico Diagnosi e Cura, S.Orsola Malpighi Hospital, Bologna, Italy; 4 Clinica Psichiatrica, San Salvatore Hospital, L'Aquila, Italy; 5 Istituto di Clinica Psichiatrica, Policlinico Consorziale Hospital, Bari, Italy; 6 Dipartimento di Salute Mentale, Azienda USL 16 Hospital, Padova, Italy; 7 Servizio Psichiatrico Diagnosi e Cura, Hospital of Adria, Rovigo, Italy; 8 Servizio Psichiatrico Diagnosi e Cura, Nuovo Regina Margherita Hospital, Roma, Italy; 9 Servizio Psichiatrico Diagnosi e Cura, Vimercate Civil Hospital, Milano, Italy; 10 Medical Department, Eli Lilly Italia, Firenze, Italy; 11 See Appendix

## Abstract

**Background:**

Few data are available on subjects presenting to acute wards for the first time with psychotic symptoms. The aims of this paper are (i) to describe the epidemiological and clinical characteristics of patients at their first psychiatric admission (FPA), including socio-demographic features, risk factors, life habits, modalities of onset, psychiatric diagnoses and treatments before admission; (ii) to assess the aggressive behavior and the clinical management of FPA patients in Italian acute hospital psychiatric wards, called SPDCs (Servizio Psichiatrico Diagnosi e Cura = psychiatric service for diagnosis and management).

**Method:**

Cross-sectional observational multi-center study involving 62 Italian SPDCs (PERSEO – Psychiatric EmeRgency Study and EpidemiOlogy).

**Results:**

253 FPA aged <= 40 were identified among 2521 patients admitted to Italian SPDCs over the 5-month study period. About half of FPA patients showed an aggressive behavior as defined by a Modified Overt Aggression Scale (MOAS) score greater than 0 Vs 46% of non-FPA patients (p = 0.3651). The most common was verbal aggression, while about 20% of FPA patients actually engaged in physical aggression against other people. 74% of FPA patients had no diagnosis at admission, while 40% had received a previous psychopharmacological treatment, mainly benzodiazepines and antidepressants. During SPDC stay, diagnosis was established in 96% of FPA patients and a pharmacological therapy was prescribed to 95% of them, mainly benzodiazepines, antipsychotics and mood stabilizers.

**Conclusion:**

Subjects presenting at their first psychiatric ward admission have often not undergone previous adequate psychiatric assessment and diagnostic procedures. The first hospital admission allows diagnosis and psychopharmacological treatment to be established. In our population, aggressive behaviors were rather frequent, although most commonly verbal. Psychiatric symptoms, as evaluated by psychiatrists and patients, improved significantly from admission to discharge both for FPA and non-FPA patients.

## Background

International studies show a growing recognition that subjects presenting with psychotic symptoms for the first time need specialized management [1]. However, few data are available about the clinical characteristics of patients at their first psychiatric admission (FPA), their management, and their subjective well-being.

In Italy, a 1978 law provides that psychiatric patients can only be admitted to hospital psychiatric wards, no other admission (i.e. to psychiatric hospitals, which were in fact abolished) shall be allowed. Very few epidemiological studies have been carried out on psychiatric in-patients since then. Acute hospital psychiatric wards are specific structures (called SPDCs in Italy, Servizio Psichiatrico Diagnosi e Cura = psychiatric service for diagnosis and management), located within general hospitals, for referral of acute patients with psychiatric-related illnesses. Patients stay in SPDCs only during the acute phase. At discharge, they usually receive therapeutic prescriptions and are no longer followed by SPDC services but by district health facilities, which are not part of general hospitals [2]. SPDCs could be the perfect setting for the study of mentally ill patients at their first presentation to hospital, although, in such emergency situations, the implementation of clinical research and epidemiology programs is rather difficult. In fact, poor information is available about clinical characteristics and behaviors of first admitted patients. Moreover, little is known about the treatments administered in SPDCs.

Aggression in psychiatric wards is common and represents a growing concern for mental health professionals, due to the increasing number of incidents [3-5]. However, recent data suggest the importance of distinguishing between different types of aggression [6,7]. Foley and co-workers showed that individuals with first episode psychosis are at high risk of demonstrating aggression at the time of presentation to hospital, but at relatively low risk of developing a behavior likely to cause injury to other people [8].

PERSEO – Psychiatric EmeRgency Study and EpidemiOlogy – is a large observational multi-center study involving 62 Italian SPDCs, aimed at assessing the prevalence and incidence of aggressive behavior in patients admitted to SPDCs and at describing the clinical features and the management of such patients. Within the admitted psychiatric patients, the PERSEO study aimed at giving a detailed description of FPA patients too. PERSEO follows EPICA, a pilot study on 728 psychiatric cases enrolled in 15 Italian SPDCs, where the Italian version of two psychometric scales, namely the Modified Overt Aggression Scale (MOAS) and the NOSIE (Nurses' Observation Scale for In-patient Evaluation), were validated [9].

This report analyzes FPA patients only, among the PERSEO population, with the objective of describing their clinical and treatment characteristics and their management inside the SPDC.

## Methods

PERSEO is a cross-sectional observational multi-center study aimed at assessing some clinical and epidemiological features of patients referring to Italian SPDCs, and in particular: their socio-demographic characteristics, the distribution of diagnoses, the prevalence of aggressive behavior, the clinical picture at presentation to SPDCs and the overall management and pharmacological treatment approach in the emergency setting. Following approval by the Ethics Committees of the participant institutions and obtaining of the patients' written informed consent, a cohort of 2521 consecutive subjects aged 18 years or more presenting to 62 SPDCs distributed throughout Italy between September 2003 and April 2004 was enrolled into the study. Patients were admitted to the study only once over the enrolment period, and subjects at their FPA were identified. Diagnosis at admission and discharge was assessed according with ICD-9 CM criteria and then grouped by diagnosis group [10].

The patients' clinical status was evaluated at admission, then for the first three days of hospital stay and at discharge or at day 30, whichever came first. Psychometric evaluations included the 24 items Brief Psychiatric Rating Scale (BPRS) [11], the Brief Symptoms Inventory (BSI) [12], and the MOAS.

The MOAS [13] has been recently validated in Italian by Margari et al. [9]. The MOAS records the onset, rates the severity of aggression episodes and can be filled out by nursing staff or physicians, on the basis of direct observation and clinical history of the 24 hours before the index visit according with previous use by other authors [8,14,15]. This scale includes four domains: verbal aggression, aggression against property, autoaggression, and aggression towards others. Each subject was rated on a scale between zero (no aggression) and four (maximum score) on each domain. The scores were weighted as described by Kay et al. [13] to calculate the total MOAS score. Only scales thoroughly filled out both at admission and discharge were considered for analysis. The prevalence of aggressive patients was then calculated as the proportion of patients receiving a total MOAS score greater then zero at admission (i.e. having shown at least one aggressive behavior within 24 hours before admission) among all patients evaluated for that scale. This dichotomization separates patients with no aggression from those ones who had any severity and type of aggression, either verbal, physical against self, others or objects.

Psychopathology was evaluated from the psychiatrist's and the patient's point of view, by means of the BPRS and the BSI respectively. The BPRS version 4.0 is a widely recognized symptomatic scale for the psychopathological evaluation of patients and was validated in Italian by Morosini et al [16]. It records the severity of symptoms on a qualitative scale with 7 levels, ranging from 'absent' to 'very serious'. The total score and the domains' scores, namely anxiety-depression, thought disorders, isolation-motor retardation, hostility-suspiciousness, hyperactivity, mania, were calculated [16]. BSI [11] is a self-evaluation scale for general psychiatric symptoms. It covers 53 items, which evaluate 9 elements: somatization, obsessive-compulsive symptoms, interpersonal sensitivity, depression, anxiety, hostility, phobic anxiety, paranoid ideation, psychoticism. The internal scores for each dimension of the scale were calculated on the scales which were fully completed.

Among the overall PERSEO population, we focused on FPA patients no older than 40. The 40 years age limit was decided in order to exclude FPA due to late onset disorders and to cognitive impairment or dementia, and also with the aim of obtaining data possibly useful for the identification of psychopathological risk factors and the characteristics of the clinical onset.

Socio-demographic and anamnestic data, life habits, reason for hospital admission, referring person, clinical evaluations, concomitant diseases, previous and ongoing psychiatric treatment, admission and discharge diagnoses and treatments administered in SPDC were recorded on case record forms [10]. Specific data were recorded for FPA patients only, such as symptoms at onset, patient management at onset, pharmacological and non-pharmacological treatment at onset and risk factors for the psychiatric disease. Project and data management other than statistics were conducted by MediData Studi e Ricerche. Data were analyzed using SAS for Windows, release 8.2. All quantitative variables were described by means, standard deviations and ranges. Absolute and relative frequency distributions were given for qualitative variables. Comparisons were performed by Student's t test for mean values, chi-square test, or Fisher exact test. When multiple comparisons were performed Bonferroni's correction was applied.

## Results

Among the 2521 patients of the overall PERSEO population, FPA patients aged < 40 years were 253 (10%). Their socio-demographic characteristics are summarized in Table 1. FPA patients were not statistically different from non-FPA patients as regards gender distribution, life habits (smoke and alcohol) and the admission status (p(chi-square test)>0.05), whereas occupational and marital status and drug abuse showed statistically different distributions (p < 0.001). Age was not compared between FPA and non-FPA patients because one of the inclusion criteria for the FPA group was "aged <= 40". Age distribution of FPA (Table 2) was not significantly different between males and females (Fisher exact test p > 0.05). However, the frequency peak was 30–34 years among women and 25–29 among men. 94 (37.2%) FPA patients were referred from a psychiatrist, 60 (23.7%) from other health professionals (physician or psychologist), 99 (39.1%) had not contacted a physician before admission to SPDC. In 215 of FPA patients, at least one psychiatric symptom was present at the onset, the most frequent being depressive symptoms (51.4%), followed by positive (38.3%), negative or cognitive (12.6%) symptoms, and manic excitement (9.3%). The onset of symptoms was slow in 13.8% of patients, gradual in 51.4%, and acute in 28.9%.

**Table 1:** Socio-demographic characteristics of FPA patients

*Gender*^*(1)*^, N (%)	FPA patients (N = 253)	Non-FPA patients (N = 2219)
Male	140 (55.3)	1118 (50.4)
Female	113 (44.7)	1101 (49.6)

*Age*, mean (SD)	30 (6.0)	45.2 (14.0)

*Occupational status*^*(2)*^, N (%)		
Employed	104 (41.1)	439 (19.8)
Housewife	30 (11.9)	298 (13.4)
Student	14 (5.5)	33 (1.5)
Unemployed	90 (35.6)	568 (25.6)
Other^(3)^	10 (3.9)	838 (37.8)
UKN	5 (2.0)	43 (1.9)

*Marital status*^*(2)*^, N (%)		
Single	139 (55.0)	940 (42.4)
Married	58 (22.9)	506 (22.8)
Widow	1 (0.4)	102 (4.6)
Divorced	14 (5.5)	257 (11.6)
UKN	41 (16.2)	414 (18.7)

*Life habits*, N (%)		
Smokers^(1)^	138 (54.6)	1283 (57.8)
Alcohol abusers^(1)^	37 (14.6)	425 (19.2)
Drug abusers^(2)^	18 (7.1)	68 (3.1)

*Admission status*^*(1)*^, N (%)		
Voluntary	206 (81.4)	1897 (85.5)
Involuntary	47 (18.6)	317 (14.3)

UKN	0 (0.0)	5 (0.2)

^(1) ^P(chi-square test between FPA and non-FPA patients)>0.05

^(2) ^P(chi-square test between FPA and non-FPA patients)<0.005

^(3) ^"Other" occupational status includes retired or unable-to-work patients

**Table 2:** Age distribution of FPA patients only, stratified by gender. Percentages are referred to the total number of FPA patients within gender

Age classes, *N (%)*	Female	Male
18–19	3 (2.7%)	5 (3.6%)
20–24	20 (17.7%)	26 (18.6%)
25–29	21 (18.6%)	38 (27.1%)
30–34	38 (33.6%)	33 (23.6%)
35–39	26 (23.0%)	35 (25.0%)
40	5 (4.4%)	3 (2.1%)

The majority of patients at their FPA had neither a known psychiatric diagnosis, nor had received previous psychiatric treatments. In particular, only 66 (24.1%) FPA patients had an admission diagnosis, as presented in Table 3 (left column). 102 (40.3%) FPA patients had received a psychopharmacological treatment before admission (Table 4, left column). This was not the case for non-FPA patients who were admitted with a known diagnosis in 73% of cases while 69% were under treatment before admission.

**Table 3:** Diagnoses at admission to hospital psychiatric wards and at discharge. The percentage is calculated on patients with an established diagnosis at admission and at discharge within each group of patients.

	**FPA patients**		**Non-FPA patients**	
**Diagnosis**	**Admission N (%)**	**Discharge N (%)**	**Admission N (%)**	**Discharge N (%)**

Affective psychosis, depression, depressive status	16 (24.2)	40 (17.4)	299 (16.6)	361 (17.1)
Schizophrenia, paranoid status, other non-organic psychosis	12 (18.2)	75 (31.5)	686 (38.2)	768 (36.3)
Substance abuse, dependence	9 (13.6)	17 (7.1)	85 (4.7)	118 (5.6)
Personality disorders	7 (10.6)	45 (18.9)	261 (14.5)	280 (13.2)
Neurotic disorders	7 (10.6)	22 (9.2)	70 (3.9)	81 (3.8)
Affective psychosis, manic episodes, excitement status	4 (6.1)	9 (3.8)	172 (9.6)	204 (9.6)
Schizoaffective psychosis	3 (4.5)	6 (2.5)	135 (7.5)	158 (7.5)
Dementia and psycho-organic syndromes	3 (4.5)	5 (2.1)	40 (2.2)	73 (3.5)
Acute stress reactions, adaptation reactions	1 (1.5)	12 (5.0)	11 (0.6)	35 (1.7)
Others	4 (6.1)	7 (2.9)	38 (2.1)	37 (1.7)

**Total N. of patients with diagnosis**	**66**	**238**	**1797**	**2115**

**Table 4:** Psychopharmacological treatment before admission to hospital psychiatric wards and at discharge. Percentages are calculated on patients on treatment, at symptoms onset (FPA only) and at discharge.

**Drug**	**At symptoms onset N (%)**	**Discharge N (%)**	
	**FPA patients only**	**FPA**	**Non-FPA**

Benzodiazepine	49 (48.0)	157 (69.2)	1447 (69.4)
Antidepressant	48 (47.1)	92 (40.5)	703 (33.7)
Conventional antipsychotic	27 (26.5)	87 (38.3)	959 (46.0)
Atypical antipsychotic	18 (17.6)	115 (50.7)	1107 (53.1)
Mood stabilizer	7 (6.9)	48 (21.1)	726 (34.8)
Anticholinergic	3 (2.9)	27 (11.9)	234 (11.2)
Other	4 (3.9)	12 (5.2)	88 (4.2)

**Total N. of patients on treatment**	**102**	**239 **	**2084**

As far as risk factors are concerned, 65 FPA patients (26%) had relatives with psychiatric disease, most commonly the mother (47.7%), followed by the father (41.5%), then brothers (21.5%), and sisters (15%). More than half of FPA patients (57%) had experienced at least one stressful event before the onset of psychiatric symptoms. Of these stressful events, 63% were emotional or family-related, 17% were financial problems, and 10% were health problems.

236 (93.3%) FPA patients were evaluable for the MOAS. About half of the FPA patients (49.2%) showed an aggressive behavior during the day before admission, more commonly a verbal one (37.7%). Aggression against properties and against other people accounted for 22.5% of patients each The aggressive behavior pattern was similar (Table 5) in the non-FPA cases (from the overall PERSEO population; p > 0.05). The most frequent diagnosis among FPA patients showing aggressive behaviors was schizophrenia (32.4% of aggressive patients). Aggressive behaviors were more common among FPA patients, whose admission was not voluntary (69%; p = 0.003), while no difference in aggressive behaviors was observed between males and females or across the different age groups in FPA patients.

**Table 5:** Prevalence of aggressive behavior in FPA patients, as expressed by MOAS. Percentages are calculated on patients valuable for MOAS, i.e. with MOAS fully compiled both at admission and discharge.

	**FPA patients (N = 236)**	**Non-FPA patients (N = 2089)**
*MOAS scores, N (%)*		
		
Verbal	89 (37.7%)	791 (37.9%)
Against Properties	53 (22.5%)	373 (17.9%)
Against Self	48 (20.3%)	318 (15.2%)
Against Other People	53 (22.5%)	424 (20.3%)
Total Score	116 (49.1%)	962 (46.1%)

During SPDC stay, a psychiatric diagnosis was established in 238 (94.10%) FPA patients (Table 3, second column) and a pharmacological therapy was prescribed to 239 (94.5%) (Table 4, second column). Psychiatric symptoms, as evaluated by psychiatrists (by BPRS) and patients themselves (by BSI), improved significantly from admission to discharge for all domains, both for FPA and non-FPA patients, as shown in Table 6. At admission, no statistically significant differences were observed for BPRS domains between FPA and non-FPA patients (p > 0.05), whereas at discharge thought disorders, hyperactivity and mania were significantly lower for FPA by comparison with non-FPA patients (p < 0.01); the remaining domains showed no statistical difference (p > 0.05). On the other hand, no significant differences were observed between FPA and non-FPA patients at admission or discharge for BSI dimensions scores (p > 0.05).

**Table 6:** Scores of main BPRS domains and BSI symptom scales at admission and discharge.

	**FPA**		**Non-FPA**	
	**Admission score **Mean (SD)	**Discharge score **Mean (SD)	**Admission score **Mean (SD)	**Discharge score **Mean (SD)
**BPRS domains (items)**				
Anxiety-Depression (2-3-5)	10.2 (4.4)	6.5 (2.8)	9.5 (4.1)	6.2 (2.8)
Thought disorders (10-11-15)	7.4 (5.0)	4.6 (2.6)	8.0 (4.8)	5.4 (3.1)
Isolation-Motor retardation (16-17-18)	7.2 (4.3)	5.1 (2.8)	7.6 (4.3)	5.6 (2.9)
Hostility-Suspiciousness (6-9-20)	9.2 (5.1)	4.8 (2.5)	8.6 (4.7)	5.3 (2.8)
Hyperactivity (19-21-24)	6.5 (3.6)	4.0 (1.7)	6.8 (3.7)	4.5 (2.1)
Mania (7-22-23)	6.1 (3.9)	4.0 (1.9)	6.8 (4.2)	4.5 (2.3)

**BSI symptom scales (items)**				
Somatization (2+7+23+29+30+33+37)/7	1.0 (0.9)	0.6 (0.6)	1.1 (0.9)	0.7 (0.7)
Obsessive-Compulsive (5+15+26+27+32+36)/6	1.3 (1.0)	0.9 (0.7)	1.4 (0.9)	1.0 (0.8)
Interpersonal sensitivity (20+21+22+42)/4	1.3 (1.0)	0.8 (0.8)	1.4 (1.0)	0.9 (0.8)
Depression (9+16+17+18+35+50)/6	1.5 (1.2)	0.9 (0.9)	1.6 (1.1)	1.1 (0.9)
Anxiety (1+12+19+38+45+49)/6	1.6 (1.0)	0.9 (0.8)	1.5 (1.0)	0.9 (0.8)
Hostility (6+13+40+41+46)/5	1.1 (0.9)	0.6 (0.7)	1.1 (0.9)	0.6 (0.7)
Phobic anxiety (8+28+31+43+47)/5	0.8 (0.8)	0.6 (0.7)	1.0 (0.9)	0.7 (0.7)
Paranoid ideation (4+10+24+48+51)/5	1.4 (1.0)	0.9 (0.8)	1.4 (1.0)	1.0 (0.8)
Psychoticism (3+14+34+44+53)/5	1.2 (0.9)	0.8 (0.8)	1.2 (0.9)	0.9 (0.8)
Total BSI	67.2 (40.8)	41.7 (32.7)	69.8 (39.5)	45.3 (33.6)

Treatment at discharge for FPA patients was mainly benzodiazepines (69% of cases), followed by atypical and typical antipsychotics (51% and 38% respectively). This pattern was not significantly different from the non-FPA cases enrolled in the study (from the overall PERSEO population), given that, at discharge, 69% and 53% of non-FPA patients were respectively on benzodiazepines and atypical antipsychotics (p > 0.05, with Bonferroni correction for 5 multiple comparisons). A higher, though not significant, proportion of non-FPA with typical antipsychotics (46%) was found. A much lower proportion of mood stabilizers was observed in FPA cases (21% Vs 35% of non-FPA cases; p < 0.001, with Bonferroni correction for 5 multiple comparisons); whereas a higher, though not significant, percentage of antidepressants (41%) was found in FPA by comparison with non-FPA patients (34%; p = 0.2, with Bonferroni correction for 5 multiple comparisons).

When psychiatric diagnosis is taken into account, benzodiazepines are still the most widely prescribed drugs in FPA patients (ranging from 60% for "substance abuse" diagnosis to 76% for personality disorders). Atypical and conventional antipsychotics are frequently given to FPA patients diagnosed with schizophrenia, paranoid status or other non organic psychoses (66% atypical, 55% typical). Atypical drugs are administered also in case of personality disorders (44%; 42% typical) or affective psychoses/depressive episode-status (45%; 25% typical). Antidepressants, as expected, were frequently prescribed to affective psychoses, neurotic disorders and patients diagnosed with acute stress/adaptive reaction (not shown).

## Discussion

To our knowledge, there are very few large epidemiological studies describing the socio-demographic and clinical characteristics as well as the aggressive behavior of first admitted patients to Italian psychiatric emergency structures, the SPDCs. The epidemiology of first episode psychoses and first psychiatric admissions is poorly understood because of the paucity of systematic studies. Yet, it is considered crucial for understanding psychiatric disorders and aggressive behaviors at presentation to hospital, in order to establish proper management [8,17,18]. A major problem is that, in emergency situations, the implementation of clinical research and epidemiology programs is rather difficult. Thus, little data are available about clinical characteristics and behaviors of first episode psychosis and FPA patients, as well as about the conduct in psychiatric wards, both in Italy and in Europe. A few years ago, the paucity of epidemiological studies carried out in psychiatric emergency structures prompted some Authors to stress the need to focus on the quality of diagnosis and management in such structures, as it happens for community structures [19]. For this study, from the overall 2521 cases collected by the PERSEO (Psychiatric EmeRgency Study and EpidemiOlogy) project – a large Italian observational multi-center study involving 62 SPDCs, aimed at assessing the prevalence and incidence of aggressive behavior in patients admitted to a SPDC and at describing the clinical features and management of such patients – we selected a sample of 253 patients aged ≤ 40 years at FPA, which represents the largest studied population of first admitted patients in Italian psychiatric emergency structures. A comparison between FPA and non-FPA patients is given here. However it is noteworthy that the comparison between groups is not the aim of the study, which is not a case-control. In fact, non-FPA patients were not selected according to any pairing criterion.

A delay in the occurrence of the first psychotic episode in females by comparison with males has been reported by several authors, at least for schizophrenia, and the role of estrogens in modulating serotoninergic function has been discussed by numerous authors [20-23]. In our population, there was no significant difference in age distribution at FPA between males and females, though the frequency peak actually occurred about 5 years later among women [30–34 years) than among men (25–29 years). The percentage of smokers was quite high among our patients (54.6%), not surprisingly, as it is well known that psychiatric patients are more vulnerable to nicotine-dependence and that people with mental illness are about twice as likely to smoke as others [24,25].

The most frequent diagnoses were depression and schizophrenia both at admission and at discharge, even though in an inverted frequency order, i.e. more depression diagnoses at admission, more schizophrenia at discharge. This is consistent with the 8-year interim results of an Irish epidemiological long term prospective study, the Cavan-Monaghan study on first-episode psychosis [19], which outlined that three are the primary diagnostic nodes at the first psychotic episode, i.e. schizophrenia spectrum psychoses, bipolar disorder, and major depressive disorder, around which there are numerous additional and overlapping diagnostic categories, that are distinct only in terms of their operational definition.

Psychiatric patients and individuals with schizophrenia in particular are reported to engage in frequent and sometimes severe acts of aggression [26-28]. However, the importance of distinguishing between different types of aggression has been pointed out [6], and recent data by Foley and co-workers, on 157 patients with a first episode of DSM-psychosis presenting to a secondary referral psychiatric service over a 4-year period, have shown that subjects with first episode psychosis are at high risk of demonstrating an aggressive behavior at the time of presentation, but at relatively low risk of engaging aggression against other people [8]. Our observational study seems to confirm that FPA patients have more commonly a verbally aggressive behavior, rather than a physical one against self, properties or other people. However, the prevalence of aggression against other people observed in our survey is higher (22.5%) than that reported by Foley (14%) [8] and by other authors. A 5-year Italian study on 1534 acute psychiatric in-patients reports 21% rate of aggressive episodes, with 7.5% prevalence of physical attacks [14], while a 3-year study on 934 psychiatric acute ward patients in Norway reports 10.5% aggressive patients [15].

In our study population, no significant difference was observed, in terms of aggressive behavior, between FPA and non-FPA patients, or between males and females. Previous data about gender differences in aggressive patients are controversial: the Norwegian study by Mellesdal et al. [15] reported no significant sex difference by total rate of aggression, but a trend for female patients to have higher rates of assaults, while Foley et al. [8] observed that males had significantly higher MOAS total scores by comparison with females, which does not exactly means a different prevalence of aggressive behavior. Concerning the possible correlation between aggression and diagnosis or first versus repeated admission, the Italian study by Grassi et al. [14] reported that most aggressive patients had a diagnosis of schizophrenia and/or delusional syndromes (55%) and previous psychiatric admissions (92%). Also in our study, the most frequent diagnosis among aggressive patients was schizophrenia, even though at a lower extent (32%), while no difference was registered in FPA versus non-FPA patients. In our study patients, a higher percentage of involuntarily admitted individuals showed at last one aggressive behavior, and this seems to definitely confirm previous observations that compulsory admission is associated with aggression [8,29,30].

The differences we have shown between the PERSEO study and other studies are difficult to interpret, as such studies differ in study design, inclusion criteria, or methods for evaluation. In fact, while Grassi et al [14] used the SOAS and defined *aggressive *all patients with a moderate or severe aggression, in the PERSEO study the MOAS was used and its total score was transformed into binary form in order to have patients with no aggression vs patients with any degree of aggression. Moreover Foley et al [8] used the MOAS, but a different classification criteria of aggressive behavior was applied: an individual was considered non-aggressive even when it scored greater than zero on the subscale for verbal aggression.

An interesting aspect to be underlined is that, despite the lack of a clearly defined diagnosis in the majority of our FPA patients before admission, almost half of them had received some kind of psychopharmacological treatment. This finding seems to mirror those of other reports from US studies [31,32] stating that many patients receiving psychopharmacological treatment have not completed a previous adequate clinical assessment and that only about half of them actually meet the diagnostic criteria for a mental disorder. During SPDC stay psychiatric diagnoses were defined and pharmacological therapy was established for almost all patients. Comparing the treatments prescribed at discharge with those received by the patients before admission, a high increase in the prescription of antipsychotics, mainly atypical, and mood stabilizers is observed. This may be explained by the fact that an adequate clinical and diagnostic assessment, as performed in the hospital setting, led to a more specific therapeutic approach. Moreover, the trends in drug use from 1993 to 2002, as reported in several American and Italian pharmacoepidemiological studies [33-36], show that there is an increasing use in atypical antipsychotics prescription, especially among psychiatric in-patients. Unfortunately, we were unable to assess the adequacy of treatment, because our study design and duration of follow-up were insufficient for such an analysis.

## Conclusion

In conclusion, our findings suggest that individuals presenting at their first psychiatric ward admission have often not undergone previous adequate psychiatric assessment and diagnostic procedures. In our population, aggressive behaviors were rather frequent, although more commonly verbal. The first hospital admission allowed diagnosis and psychopharmacological treatment to be established in almost the totality of patients.

The strength of this study lies in the relatively large number of FPA patients, although their description is limited to an Italian sample of psychiatric patients. This particular setting might be the reason of some differences we have found in treatment and patient management. Therefore, future surveys on psychiatric ward patients should address two further objectives: first, the identification of possible patient-staff interactions and of situations favoring or precipitating the aggressions; secondly, the assessment of the adequacy of the established therapy.

## Authors' contributions

AB, RB, GB, MC, FM, LM, RR, FR, AS are the members of the PERSEO study Advisory Board. They all contributed to the study protocol design, enrolment, interpretation of analyzed data and to the review of this paper.

SF, AR, MS are current employees of Eli Lilly Italy, i.e. the study sponsor. They contributed to the interpretation of analyzed data and to the writing and review of this paper. Finally, the members of the PERSEO study group contributed to patient's enrolment.

## Appendix: the PERSEO study group

The following centers have collaborated to the study:

▪ Barale F, Bonzano A, Scioli R – Neurol. Inst. of Mondino Pavia

▪ Bellomo A, De Giorgi A, Cammeo C – Osp.Riuniti Hospital Foggia

▪ Cao A, Zara B – San Francesco Hospital Nuoro

▪ Conforti I, Chillemi C – Psychiatric Department Parma

▪ Dagnino L, Ponzoni M – Ospedali Riuniti Hospital Bergamo

▪ Della Pietra F, Benettazzo M – Azienda USL 16 Hospital Padova

▪ Esposito V, Sposito M – Psychiatric Department Palermo

▪ Fato M, Signorello G – Hospital department of Ponente Genova

▪ Fiorenzoni S, Singali A – Ponte Nuovo Hospital Firenze

▪ Margari F, Sicolo M – Policlinico Consorziale Hospital Bari

▪ Martino C, Leria G – Santa Croce Hospital Torino

▪ Tavolaccini L, Nigro G – Martini Hospita lTorino

▪ Russo V, La Rovere R – SS. Immacolata Hospital Chieti

▪ Righi R, Mazzo M – Hospital of Adria Rovigo

▪ Rocchetti R, De Martiis L – Umberto I Hospital Ancona

▪ Rodighiero S, Morello M – Hospital of Monselice Padova

▪ Vescera M, Pisciotti D G. – Iannelli Hospital Cosenza

▪ Villari V, Barzegna G – Molinette S. G. Battista Hospital Torino

▪ Annicchiarico V, Cosmai MG – Hospital of Venere Bari

▪ Rossi G, Baraldi E C. – Poma Hospital Mantova

▪ Casacchia M, Ruggiero D – San Salvatore Hospital L'Aquila

▪ Galimberti P, Fellini F A. – Angelucci Hospital Roma

▪ Francobandiera G – Civil Hospital Sondrio

▪ Gaspari D, Turati – SSS. Trinità Hospital Novara

▪ Matacchieri BG. Moscati – Hospital Taranto

▪ Mautone A, Casale M – Hospital of Sant'Arsenio Salerno

▪ Mellado C, Scaramelli B- L. Sacco Hospital Milano

▪ Filippo A, Miccichè M – Beato Angelo Hospital Cosenza

▪ Minervini L, Banzato C – Azienda USL 16 Hospital Padova

▪ Orengo S, Alisio G – San Paolo Hospital Savona

▪ Picci RL, Venturello S – S. Luigi Gonzaga Hospital Torino

▪ D'Aloise A, Vaira F- S. Timoteo Hospital Campobasso

▪ Boccalon RM, Cavrini L – Sant' Anna Hospital Ferrara

▪ Cogrossi S, Prato K – Osp. Maggiore Hospital Cremona

▪ Cremonese C, Menardi A – Azienda Hospital Padova

▪ Parisi M, Mentastro C – Umberto I Hospital Enna

▪ Prosperini P, Binda V – Magg. della Carità Hospital Novara

▪ Romano G, Materzanini A – Mellino Mellini Hospital Brescia

▪ Crudele A, Stella G – Hospital of Barletta Bari

▪ Petio C, Fuà B – Ottonello Institute Bologna

▪ Laich L, Miori M – Hospital department of Arco Trento

▪ Salteri A, Catania G – Vimercate Civil Hospital Milano

▪ Achena M, Fara FM – Hospital of Sassari Sassari

▪ Padoani W, Compagno S – Hospital of Conegliano Treviso

▪ Ballerini A, Pecchioli S, Moretti S. – S.M.N. Hospital Firenze

▪ Bacchi L, Vicari E – Hospital of Partinico Palermo

▪ Arvizzigno C, Minunni P – F. Iaia Hospital Bari

▪ Rossi E, Zaiti MF – L. Pierantoni Hospital Forlì Cesena

▪ Boncompagni G, Selleri M S. – O. Malpighi Hospital Bologna

▪ Minnai GP, Loche AP – San Martino Hospital Oristano

▪ Russo F, Antonucci A – Nuovo R. Margherita Hospital Roma

▪ Chiurco L, Amendola R – G. Compagna Hospital Cosenza

▪ De Giovanni MG, Martano A – V. Fazzi Hospital Lecce

▪ Borsetti G, Santone G – Umberto I Hospital Ancona

▪ Pettolino AR, Lisanti F – Umberto I Hospital Foggia

▪ Parodi A, Ciammella L, Botto G. – Villa Scassi Hospital Genova

▪ Gillotta S, Florio G – Cannizzaro Hospital Catania

▪ Fiore F, Santangelo E – A. Landolfi Hospital Avellino

▪ Fucci G, Ricci M – Psychiatric Department Ravenna

▪ Ciaramella A, Della Porta A – S. Sebastiano M. Hospital Roma

▪ Sittinieri M, D'Asta L – Paternò Arezzo Hospital Ragusa

▪ Triolo S, Spatola A – ARNAS Civil Hospital Palermo

▪ Frediani S, Rossi A, Macchi S, Giovannini L, Germani S, Fabbri L – Eli Lilly Italia, Florence, Italy

▪ Fiori G (project leader), Sala S (clinical project manager assistant), Sgarbi S (clinical project manager), Simoni L (statistics), Zanoli M (clinical data manager) – MediData Studi e Ricerche, Modena, Italy.

## Pre-publication history

The pre-publication history for this paper can be accessed here:


